# Novel Mitochondrial Substrates of Omi Indicate a New Regulatory Role in Neurodegenerative Disorders

**DOI:** 10.1371/journal.pone.0007100

**Published:** 2009-09-18

**Authors:** Felicity Johnson, Michael G. Kaplitt

**Affiliations:** Department of Neurological Surgery, Cornell University, Weill Medical College, New York, New York, United States of America; National Institutes of Health, United States of America

## Abstract

The mitochondrial protease OMI (also known as HtrA2) has been implicated in Parkinson's Disease (PD) and deletion or protease domain point mutations have shown profound neuropathologies in mice. A beneficial role by OMI, in preserving cell viability, is assumed to occur via the avoidance of dysfunctional protein turnover. However relatively few substrates for mitochondrial Omi are known. Here we report our identification of three novel mitochondrial substrates that impact metabolism and ATP production. Using a dual proteomic approach we have identified three interactors based upon ability to bind to OMI, and/or to persist in the proteome after OMI activity has been selectively inhibited. One candidate, the chaperone HSPA8, was common to each independent study. Two others (PDHB subunit and IDH3A subunit) did not appear to bind to OMI, however persisted in the mito-proteome when OMI was inhibited. Pyruvate dehydrogenase (PDH) and isocitrate dehydrogenase (IDH) are two key Kreb's cycle enzymes that catalyse oxidative decarboxylation control points in mitochondrial respiration. We verified both PDHB and IDH3A co-immunoprecipitate with HSPA8 and after elution, were degraded by recombinant HtrA2 *in vitro*. Additionally our gene expression studies, using rotenone (an inhibitor of Complex I) showed *Omi* expression was silenced when *pdhb* and *idh3a* were increased when a sub-lethal dose was applied. However higher dose treatment caused increased *Omi* expression and decreased levels of *pdhb* and *idh3a* transcripts. This implicates mitochondrial OMI in a novel mechanism relating to metabolism.

## Introduction

Omi (HtrA2/PARK13) was originally identified as a homologue of a bacterial protease HtrA [1]. In mammalian cells, the nuclear-encoded OMI resides in the inter-membrane space (IMS) of mitochondria as active, mature (32–36 kD) and pre-processed 50 kD forms. A second pool of OMI resides on the ER [2]. OMI activity is induced by stress, causing auto-catalysis at A^133^ [3]. This facilitates release into the cytosol, removing a trans-membrane domain and exposing a domain that binds with the most studied OMI substrate, the anti-apoptotic factor XIAP. OMI precipitates apoptosis by binding and degrading XIAP, causing the release and subsequent activation of caspases [4]. In addition it's protease activity in the cytosol may contribute by selective turn-over of key proteinaceous substrates [4], [5] (for a review please see [6]).

Our interest is in the mitochondrial role of OMI in neurodegenerative disorders. OMI is a homo-trimer, where each monomer consists of a MLS, a small trans-membrane region, the AVPS sequence (for XIAP interaction), the protease domain and the PDZ domain (governing substrate interaction) [4], [5], [7]–[15]. Mitochondrial dysfunction is coincident with PD [16] and in humans Omi deficiencies have been found in PD patients [17]. In addition OMI has been consistently detected in Lewy Bodies (LB) - the intracellular aggregates that represent one of the hallmarks of PD and LB Dementia [18]. Two other studies have strongly contested any genetic implication of Omi with PD [19], [20], nevertheless animals with a lack of this protease show a profound neuropathology. The *mnd*2 mutation, that spontaneously arose as an autosomal recessive disorder was identified as a mis-sense mutation (S276C) in the protease domain [21], [22]. It manifest as abnormal spinal motor neuron morphology and muscle denervation leading to muscle atrophy and progressive paralysis [21]. Neurodegeneration of striatal neurons occurred around 3 weeks of age, accompanied by astrogliosis and the activation of microglia with animals rarely living past 40 days p.n. [22]. The protease activity of Omi was greatly reduced in the tissues of *mnd*2 mice, albeit restored by a partial deletion in the PDZ domain, indicating substrate binding may have been a root cause. Additional studies using targeted deletions of the mouse Omi gene (*Prss25*) developed a parkinsonian phenotype with loss of striatal neurons [23]. Simultaneous deletion of another predominant IAP-binding protein did not alter the animal or cellular phenotype, indicating it was the protease activity of Omi that was beneficial, rather than the IAP interaction. With respect to its mitochondrial function, recent reports have given some clues. The loss of Omi in the brain was shown to cause an accumulation of unfolded mitochondrial proteins and ROS, which could signal the increased expression of the transcription factor CHOP. CHOP expression is a part of the Integrated Stress Response (ISR) and was seen to be increased in PD patients [24]. Although most studies place the role of OMI in the maintenance of the mito-proteome and the avoidance of ROS accumulation, there are few known mitochondrial substrates. Extensive studies have been done to discern the cytosolic substrates [6] with the only known mitochondrial substrate HAX-1 (an anti-apoptotic factor) keeping the focus upon OMI as a death effector [25]. Recently mitochondrial APP was reported to be a substrate, cleaved by either mature or immature OMI in the mitochondrion, furthering the importance of this protease in neuronal function [26].

OMI, as a substrate of other enzymes such as Akt, Rhomboid-7 and Pink-1 [27]–[29], has led to proposed regulatory mechanisms for this serine protease. In particular, mutations in the kinase Pink-1/PARK6 are a known cause of familial PD, with aberrant mitochondrial morphology clearly exhibited in mammalian cells [30]. Pink-1 has been shown to interact with OMI and phosphorylate several key amino acid residues leading to increased OMI activity [28]. Additionally Parkin/PARK2, another gene with mutations known to cause familial PD, had been shown to rescue Pink-1 mutations [30]. All of this has led to a multi-factorial mechanism for maintaining mitochondrial integrity, where the role of OMI was downstream of Pink-1 (which in turn was upstream of Parkin), but was independent of Parkin [29], [31], [32]. While no consensus has been reached, it appears likely that both Omi and Parkin act via parallel pathways, with respect to Pink-1, in maintaining mitochondrial integrity. The added importance of the ATP-dependent mitochondrial Rhomboid-7 was shown by its ability to co-immunoprecipitate and cleave both PINK1 and OMI into mature forms that are no longer membrane-tethered [29]. In this way Rhomboid-7 (or its human homologue PARL) could be seen as an activator of both enzymes during times when ATP was not limiting.

Our main interest was in discerning the mitochondrial substrates of OMI. In this study, we used two approaches (functional and structural) to discover three novel substrates, one of which is the chaperone for the other two, which are subunits of regulatory Kreb's cycle enzymes. We then studied the coincidental expression of these players under lethal and sub-lethal conditions. From our results, we hypothesize a novel mechanism for the role of Omi in the mitochondrion, which may account for its beneficial role and contribution to neurodegenerative processes, and move Omi beyond protein quality control.

## Results

### Degradomic approach using the mito-proteome and OMI inhibitor ucf-101

**Figure 1:**
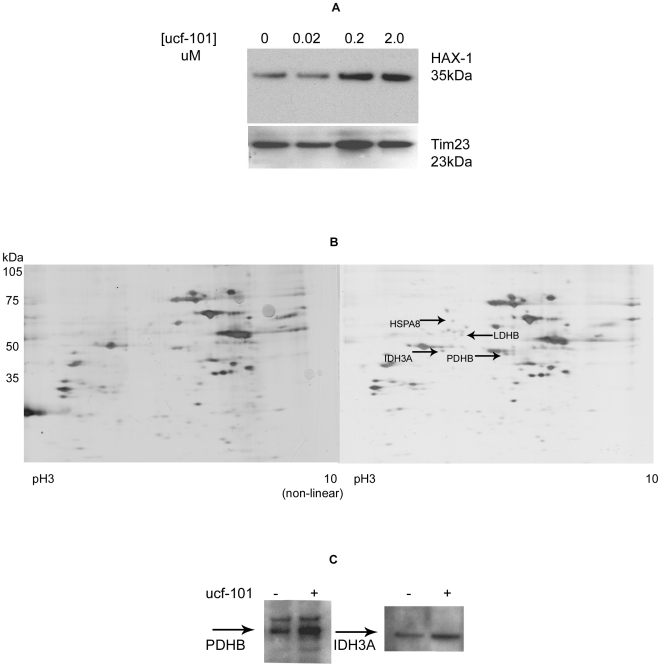
Inhibition of HAX-1 degradation by native OMI in HEK293 cells. (A) Immunoblot of mitochondrial proteins showing increased persistence of HAX-1 with increased concentration of the OMI inhibitor ucf-101. (TIM23 is shown as a loading control). (B) Silver stained IEF/2D gel of mitochondrial proteins present after isolated mitochondrial samples were divided into two equal parts and incubated in the presence or absence of ucf-101. Arrows indicate species that persisted when the inhibitor ucf-101 was included. (C) Immunoblot of mitochondrial samples validating the increased persistence of PDHB and IDH3A in the presence (+) of ucf-101.

Differential proteomic samples were generated by incubating mitochondria, isolated from HEK293 cells, in the presence or absence of the serine protease inhibitor, ucf-101 for 2H under aerobic conditions. Isolated mitochondria were used because a previous report had shown non-specific cellular effects of ucf-101 [33]. Inhibitor concentrations were chosen from the literature and following incubation trials with immunoblotting to detect persistence of the OMI substrate, HAX-1 (figure 1A) [34]. IEF/2D electrophoresis of concentrated mitochondrial proteins discerned several protein species that persisted in greater amounts when OMI was inhibited (figure 1B). Several were arbitrarily chosen, manually cored and sent for analysis via in-gel trypsin digestion and MS-MS (Rockefeller Core). This method has limitations in that it represents a small sub-set of proteins that were potentially cleaved by OMI. The table of those identified samples that were of particular interest to us (Table 1) included subunits of two Kreb's Cycle enzymes (PDHB and IDH3A)). The third identified protein was HSPA8 (also known as heat shock 70 kDa protein 8, HSP73, HSPA10, constitutive heat shock protein 70, heat shock cognate protein, 71-kDa, for nomenclature guidelines see [35]). We then verified these proteins by immunoblot detection (normalized for total mitochondrial protein) as shown in figure 1C.

**Table 1:** Proteins identified via MS-MS.

	**Functional Proteomic Approach**
accession	gene and protein name
P11142	HSPA8 Heat shock cognate 71 kDa protein (Heat shock 70 kDa protein 8)
P11177	PDHB Pyruvate dehydrogenase E1 component beta subunit, mitochondrial precursor (EC 1.2.4.1)
P50213	IDH3A Isocitrate dehydrogenase [NAD] subunit alpha, mitochondrial precursor (EC 1.1.1.41)
P07195	LDHB L-lactate dehydrogenase B chain (EC 1.1.1.27)
P08107)	HSPA1 Heat shock 70 kDa protein 1
	**Structural (substrate trap) Proteomic Approach**
P11142	HSPA8 Heat shock cognate 71 kDa protein (Heat shock 70 kDa protein 8)
P30101	PDIA3 N-terminal of the glycoprotein chaperone ERp57 (chain A) (EC 5.3.4.1)

Table depicting the identified proteins from spot excision of the silver-stained IEF/2D gels in the functional and substrate trap proteomic approach.

### Structural “entrapment” approach using the mito-proteome and tagged OMI

**Figure 2:**
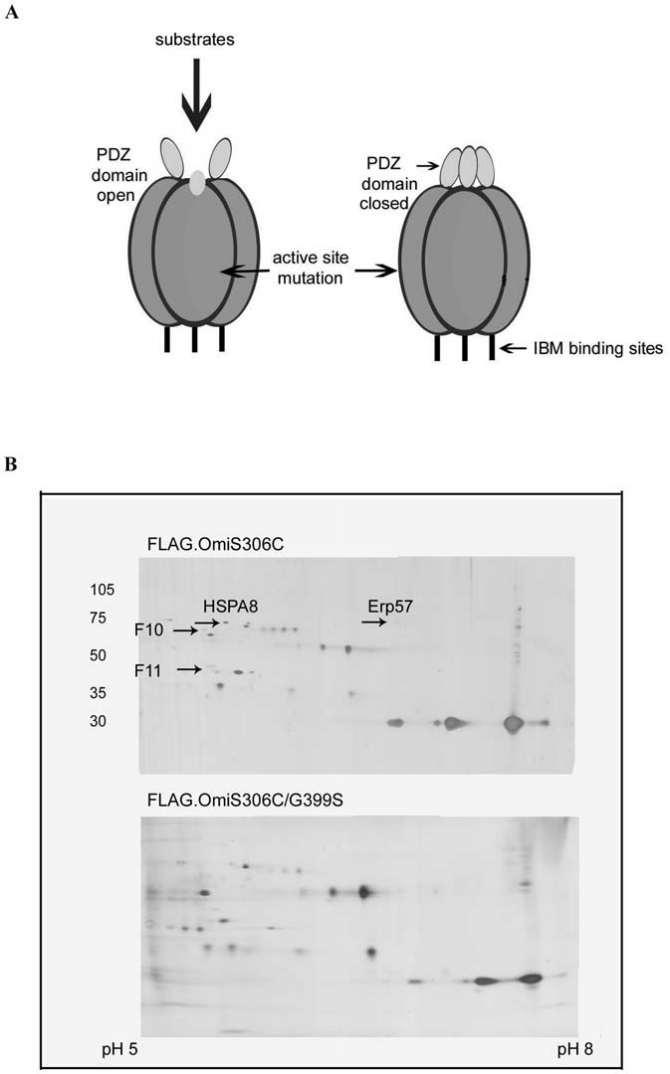
Results from the entrapment approach used to identify mitochondrial proteins interacting with OMI. A cartoon depicting OMI as a trimer with the regulatory PDZ domain (A). A representative silver stained IEF/2D gel of mitochondrial proteins from HEK293 cells transfected with FLAG-OMI mutant and immunoprecipitated with anti-FLAG beads (B). Arrows indicate the species that were unique to the OMI306 mutant but not common to both OMI.S306C and OMI.S306C/G399S.

Proteins that directly interacted with OMI, were isolated and identified using an approach that exploited the trimeric shape of OMI to “trap” substrates. A similar approach had been used with the large, bacterial multimeric protease ClpP/X [36]. We enriched for two pools of proteins that were co-immunoprecipitated with FLAG-tagged OMI protease mutants. These OMI mutants had either an inactive site, or the same active site mutation accompanied by a mutated PDZ domain (figure 2A). The first mutant OMI would entrap substrates within the trimer's cleft, whereas the second mutant could not due to a mutation in the PDZ domain. This mutation disallowed the conformational change giving substrate access. Any species isolated via this double mutant represented those binding to the OMI trimer either non-specifically, or specifically (but not as a substrate for cleavage). IEF/2D electrophoresis of concentrated mitochondrial proteins and comparison of protein spots detected in the former (OMI S306C/FLAG) but not the latter (OMI S306C.G399S/FLAG) showed several unique species. These were manually cored and identified via in-gel trypsin digestion and tandem MS (Cornell, Ithaca Core Facilities). A representative gel for both mutant pull-down experiments shows some of the samples sent for identification in figure 2B. The combined results are tallied in table 1. These results revealed that the heat shock cognate protein was common to both the FLAG/IP pull-down approach and the ucf-101 studies. This provided strong evidence that HSPA8 was affected by the protease activity of OMI and directly interacted with the enzyme.

### Validation of candidates' ability to interact with HSPA8 and be degraded by HtrA2

**Figure 3:**
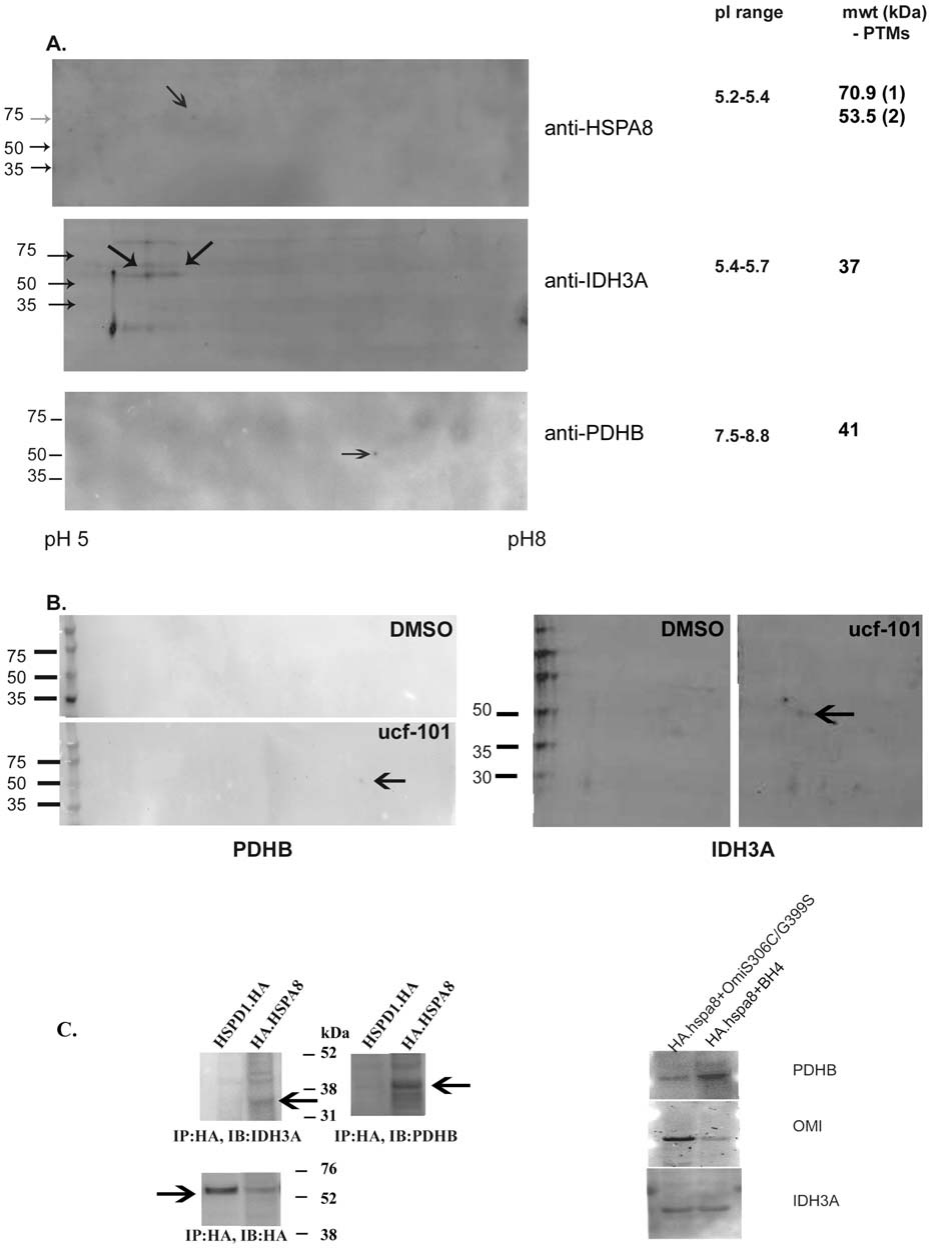
Validation of candidates' ability to interact with HSPA8 and be degraded by HtrA2 Representative immunoblots (A) of IEF/2D separated, post-IP mitochondrial proteins from HEK293 cells expressing the FLAG.OMI.S306C mutant. Arrows indicate immunoreactive spots according to the antibody used. (B) Samples were also incubated in the presence/absence of ucf-101 with recombinant HtrA2/OMI and separated and immunoblotted as above. Arrows indicate the species that correlated to the results in (A) and that persisted in the presence of ucf-101, but were absent without inhibition of OMI. (C) Immunoblots of anti-HA immunoprecipitated proteins. Arrows indicate immunoreactive spots according to the antibody used. HA.HSPD1 was used as a mitochondrially-targetted HA-tagged protein control.

We investigated co-precipitation of PDH and IDH using specific HSPA8 antibodies. Previous studies in a non-neuronal system, had linked PDH with HSP70, showing co-immunoprecipitation of PDH when anti-HSP70 was used, and *vice versa* [37]. Mitochondrial isolates were used to eliminate cytosolic interactors and, in the case of IDH, cytosolic isozymes. The results, showed small but detectable amounts of each interactor with the endogenous HSPA8 (figure 3A). In all cases the protein species identified appear slightly larger than predicted, but within the appropriate pI range. We then tested whether OMI could digest PDH and IDH subunits. An *in vitro* assay was used, where the co-IP eluted samples were divided into two parts, each being incubated with recombinant HtrA2/Omi in the presence or absence of 40 uM ucf-101. Figure 3B shows a representative immunoblot probed for PDHB and IDH3A. In both cases the presence of the OMI inhibitor ucf-101 allowed the persistence of both species of endogenous HSPA8 interactors from isolated mitochondria. To confirm this interaction using exogenous HSPA8, we transfected HA-tagged HSPA8 with OmiS306C/G399S over-expression or the empty vector (BH4). As a control we included another mitochondrial chaperone HSPD1. The resulting hetero-trimers of OMI (formed with both the endogenous active and introduced inactive monomers) were a means of increasing detection under these conditions. A representative immunoblot of the pull-down (Figure 3C) showed all three species were detected. Comparatively greater amounts of OMI were seen when the mutant OMI was over-expressed as expected, due to the antibody not distinguishing between the endogenous and exogenous forms. Surprisingly less PDHB and more IDH3A were seen in the total protein normalized samples from the double mutant. More detailed studies on the nature of each of the interactions are required in order to explain this.

### Relevance to neuronal damage

**Figure 4:**
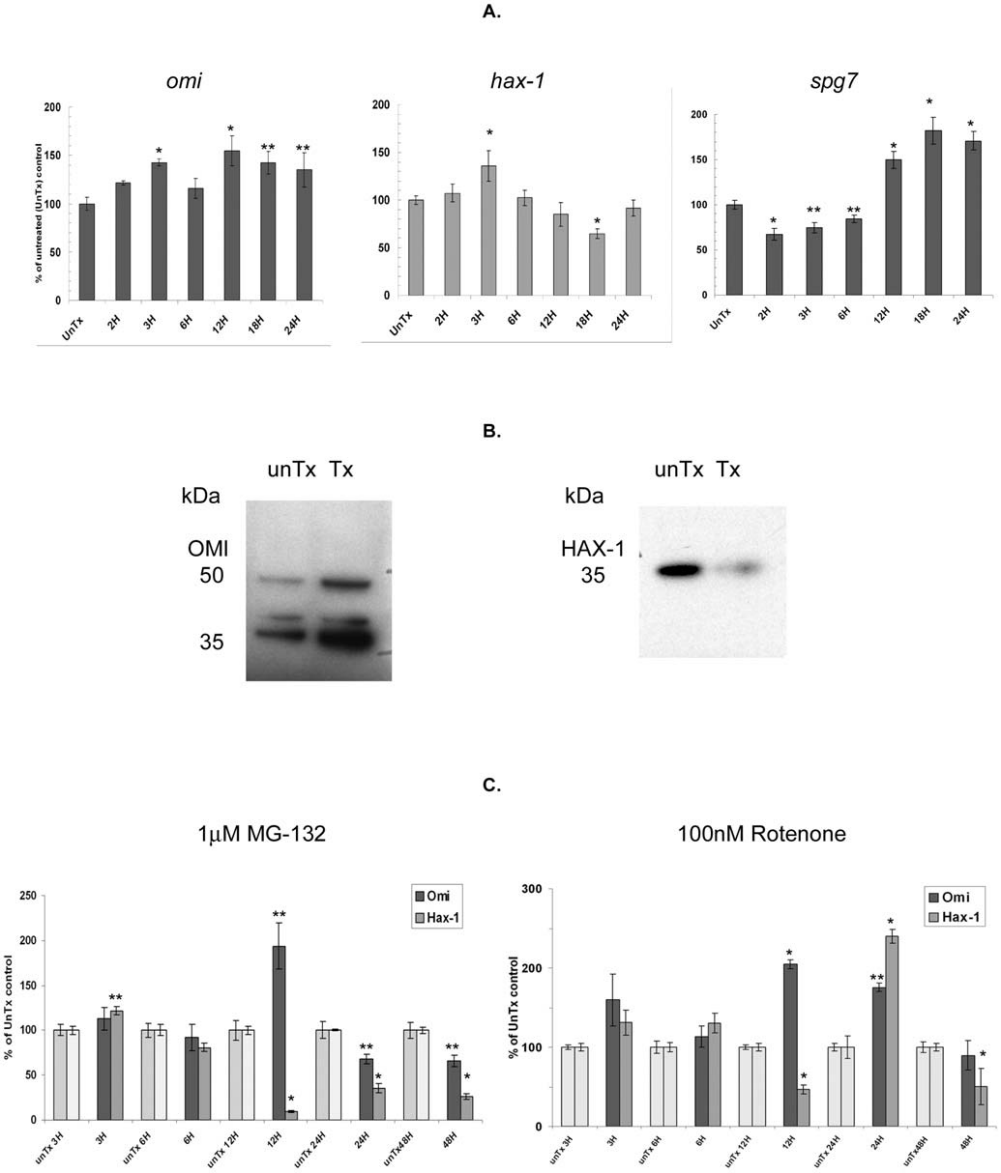
Gene expression studies of *omi*, *hax*-1 and *spg*7 (paraplegin) in SH-SY5Y cells treated with 100 µM 6OHDA (A). Expression is normalized for β-actin and shown as a percentage of the untreated control (UnTx). (B) Representative immunoblots showing the level of OMI and HAX-1 in untreated and 6OHDA-treated SH-SY5Y cells. (C) Gene expression studies of *omi* and *hax-*1 in SH-SY5Y cells treated with the proteasome inhibitor MG132 (1 µM) or Complex I inhibitor Rotenone (100 nM). Expression is shown as a percentage of the untreated control (UnTx) where all data is the Mean±SEM: n≥3, and where * indicates P≤0.005 and ** indicates P≤0.05 (using two tailed t-test analysis).

Fluctuations in gene expression often reflect adaptive changes in the proteomic profile of the cell. To further evaluate OMI and these novel interactors, we chose to study their gene expression in during neuronal stress. We investigated *omi, pdhb, hspa8*, and *idh3a* gene expression in an established cell model for PD, with all samples normalized to β-actin. Human neuroblastoma (SH-SY5Y) cells treated with the dopamine neurotoxin 6OHDA (100 µM) showed an increase in *omi* gene expression above the untreated control by 2 hours (figure 4A). This reached a maximum of 143% at 3 hours and was maintained above control levels for up to 24 hours post-treatment. Mitochondria isolated at 24 hours post-treatment showed an increase in OMI protein relative to its substrate HAX-1 (figure 4B same immunoblot comparison) indicating the increased gene expression led to greater amounts of functional protease. We also found altered *hax*-1 expression. As with *omi*, *hax*-1 showed a similar, early increase in expression although this lagged *omi* expression by at least one hour (figure 4A). Maximal *hax*-1 expression (136%) level was also seen at 3 hours however, unlike *omi* expression, was not maintained. In order to discern if this were an *omi*-specific effect, the gene expression of another mitochondrial protease, paraplegin (*spg*7), was tested (figure 4A). Its expression appeared to have a bi-phasic response to 6OHDA treatment and, unlike *omi*, was reduced in the first 6 hours of receiving treatment. Expression dropped to approximately two thirds of the untreated control within the first 2 hours, with gradual increase to 75% and 85% at 3 and 6 hours respectively. The second phase showed an increase to 150% and as high as 182% of control during the next 12 hour period. These dissimilar patterns between the two proteases, showed the 6OHDA treatment effect on *omi* was gene-specific.

Further studies showed this pattern was also toxin-dependent and not a general stress response. When either the proteosome inhibitor MG-132 (1 uM) or the mETC inhibitor, rotenone (100 nM) were used, neither *omi* nor *hax-1* gene expression followed the same pattern as during 6OHDA toxicity (figure 4C). MG-132 toxicity resulted in an increase in *omi* gene expression at 12 hours post-treatment. Other time points that differed significantly from the untreated control were at 24 and 48 hours, when *omi* expression was reduced. The overall pattern of *omi* expression during inhibition of cytoplasmic protein turnover by MG-132 was in stark contrast to that seen with 6OHDA treatment. Likewise rotenone treatment showed an early increase in *omi* gene expression above that seen with the untreated control. This fluctuated greatly over the 48 hour time period, returning to control levels by 48 hours, with maxima seen at 12 and 24 hours. Comparison between MG-132 and rotenone treatments showed only two time points with a common *omi* expression response namely, the control-level expression seen at 6H post-treatment, and the coincidental peak in expression at 12 hours. When we compared the peak with *hax*-1 expression at 12 hours, rotenone and MG-132 treatment both gave a large increase in *omi* expression (205% and 194%), coinciding with a dramatic decline in *hax*-1 expression (47% and 9%) respectively. By contrast, neither of these changes was seen at 12 hours with 6OHDA treatment, where *hax*-1 expression had already returned to control levels.

**Figure 5:**
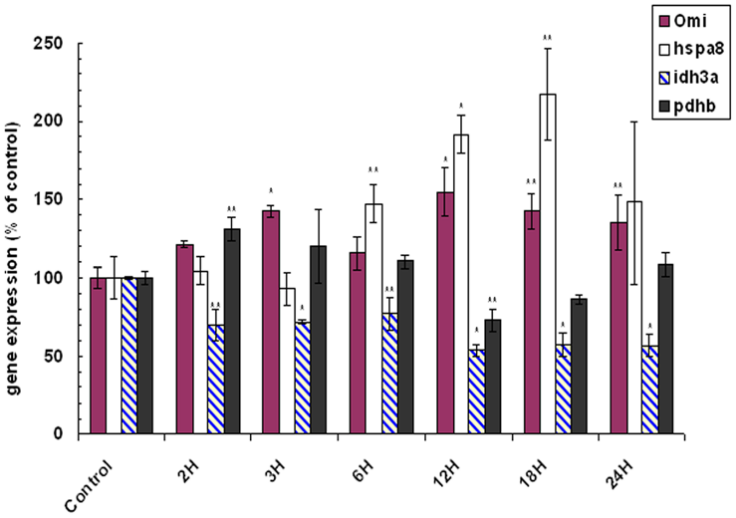
Gene expression studies of *omi*, *idh3a, pdhb* and *hspa*8 in SH-SY5Y cells treated with 100 µM 6OHDA. Expression is normalized for β-actin and shown as a percentage of the untreated control (UnTx) where all data is the Mean±SEM: n≥3, and where * indicates P≤0.005 and ** indicates P≤0.05 (using two tailed t-test analysis).

The expression of our novel OMI interactors also altered with the neurotoxin treatment. Quantitative PCR results showed that initially the level of *hspa8* remained at the same level as the untreated control cells, but after 3 hours was increased to a greater magnitude than that seen with *omi* (Figure 5). Omi expression was elevated up to 143% by 3 hours, whereas *hspa8* expression did not peak until 12–18 hours at which point it was more than 217% of the untreated control. Gene expression of both *idh3a* and *pdhb* was diminished. After an initial increase at 2 hours, the expression of *pdhb* stayed mostly at untreated levels with a drop down to approximately 73–86% at 12 and 18 hours post-treatment, whereas *idh3a* had declined to 70% of the untreated gene expression levels by 2 hours and remained at this or lower for the remainder of the sampling period. This result implied that there was some degree of regulation in the expression levels of these subunits of two Kreb's Cycle enzymes, in response to the oxidative stress resulting from the 6OHDA treatment. However the general, decreased expression of *pdhb* and *idh3a* while *omi* expression was increasing, warranted further investigation.

### Further gene expression studies

**Figure 6:**
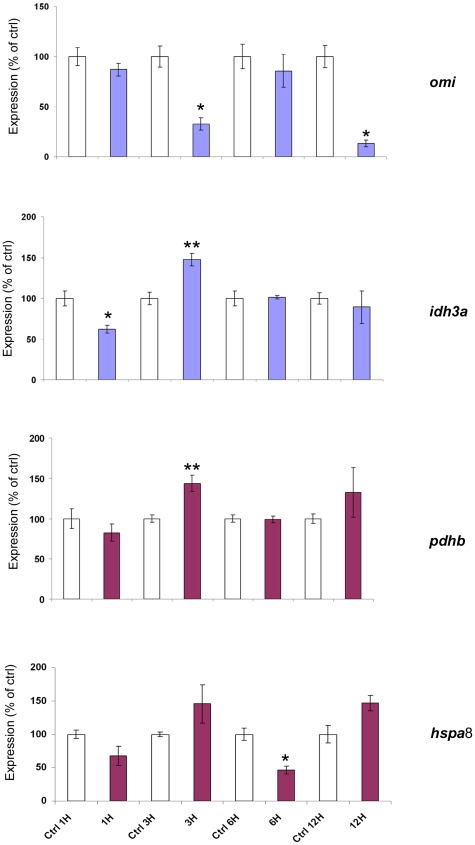
Gene expression studies of *omi*, *idh3a, pdhb* and *hspa*8 in HEK293 cells treated with 10 nM Rotenone. Expression is normalized for β-actin and shown as a percentage of the untreated control (UnTx) where all data is the Mean±SEM: n≥3, and where * indicates P≤0.005 and ** indicates P≤0.05 (using two tailed t-test analysis).

The 6OHDA cell model used above represented severe, neurotoxic conditions. We also looked at *omi*, *pdhb, idh3a* and *hspa8* gene expression during conditions when oxidative phosphorylation (OXPHOS) capacity was challenged, but not severely compromised. During sub-lethal inhibition of complex I by rotenone, the pressure from continued Kreb's cycle turn-over would be most apparent at complex II. Our novel OMI substrates included subunits of mitochondrial (mt)IDH (catalyzing the immediate step before succinate dehydrogenase) and PDH (supplying acetyl CoA to the Kreb's cycle). We hypothesized the expression of these interactors would be good barometers for change under sub-lethal (10 nM) rotenone treatment. Gene expression patterns of the three identified interacting proteins in HEK293 cells (Figure 6) showed *omi* expression began decreasing at 1 hour post-treatment, after which it decreased markedly at 3 and 12 hour time points (33% and 14% respectively). The 6 hour sample showed expression not unlike the uninhibited control. By contrast *pdhb* expression was increased at those times when *omi* was maximally diminished (3H and 12H). Likewise, *idh3a* showed its greatest expression at 3 hours (148%), after an initial decrease at 1 hour. Expression of the chaperone *hspa*8 showed a trend towards increased expression at 3 hours. However by 6 hours, expression was significantly diminished to less than half of the uninhibited control, just as *omi* was increasing back up to control levels. At 12 hours *hspa*8 expression was increased again, while *omi* expression was diminishing. The overall trend seen with sub-lethal rotenone treatment was to cause increased or decreased expression of the three OMI substrates, when *omi* expression itself was decreasing or increasing respectively.

## Discussion

Our analysis of the mitochondrial degradome (in the presence/absence of OMI inhibitor ucf-101) allowed the discovery of novel OMI substrates important to aerobic respiration. The third substrate was a specific chaperone, HSPA8. We also identified the same chaperone via the “entrapment” approach, using FLAG-tagged mitochondrial OMI. This allowed pursuit of the relationship between the four proteins. Previous work by others in different cell types had shown PDH was a binding partner with HSP70. It is worth noting that HSPA8 is a constitutive heat shock protein, with sequence similarity to the stress-inducible HSP70. Both are members of a large family of heat shock proteins. Despite their abundance in the cytosol, and in association with and in mitochondria, we did not identify HSP70 or any other related heat shock family members beyond the homologous sub-regions of the HSPA8 sequence.

Using isolated mitochondria, we have shown the HSPA8-specific antibody was able to co-immunoprecipitate small amounts of IDH3A and PDHΒ. Our studies employed IEF/2D separation due to the small amounts of protein involved, as well as the weaker epitope-interaction by the HSPA8 antibody. In doing so, we have established a new, potential role for endogenous HSPA8, distinct from its many cytosolic roles, as being that of transporting IDH3A and PDHΒ to the mitochondrion. Additionally, when the immunoprecipitated species was incubated with recombinant HtrA2 there was degradation unless the inhibitor ucf-101 was present. We verified the endogenous HSPA8 results using HA-tagged HSPA8 co-immunoprecipitation studies. All three species (OMI, PDHB and IDH3A) were co-precipitated to varying degrees. We concluded that both subunits can be bound by HSPA8 and all three proteins may be degraded by OMI at or inside the outer membrane of the mitochondrion. More direct evidence of this physical interaction was seen in a report investigating Lewy Bodies (LBs), the proteinaceous aggregates formed in PD brains. Clearly visible within tissue sections, LBs are mostly comprised of α-synuclein, and are a histopathological hallmark of PD. Using laser capture micro-dissection to study the other protein components in LBs from PD brains, HSPA8 was identified as one of the major components within these aggregates [38]. Additionally Kawamoto et al. showed increased OMI present in inclusion bodies of neural cells in the tissue of patients with α-synucleinopathies [18]. These two independent studies establish that aberrant levels of both proteins (OMI and HSPA8) occur in PD brains, supporting our results that show the relationship between them.

Establishing the conditions where this relationship is relevant and how it could be tested was made difficult by the roles of both OMI and HSPA8. Each may be located or re-located to two other major compartments of the cell, namely the cytosol and endoplasmic reticulum. In addition, the death effector role of OMI disallows over-expression studies.

In this current study, and in relation to PD, we have shown neurotoxicity (caused by 6OHDA) increased *omi* transcription in neuroblastoma cells, and with this came a coincidental, relative decrease in the novel OMI substrates. We believe this to be an initial, protective role of mitochondrial OMI. Under conditions of acute 6OHDA toxicity, the increased ROS production via OXPHOS establishes a strong incentive to shift away from using the Kreb's cycle for anabolic components and energy production. Under these conditions, the need to limit ROS production causes an increase in OMI, so it might degrade the incoming regulatory subunits of Kreb's cycle enzymes PDH and mitochondrial IDH. While this may initially be protective, continued stress would reverse OMI's beneficial role, making the release of activated OMI and other death-effectors from the damaged mitochondrion inevitable.

Poisoning by 6OHDA provided an acute and severe condition in which to study *omi* expression. Further from this, we chose conditions where OMI activity would not be ideal, namely when OXPHOS was restricted (in the absence of excess oxidative damage) and substrate availability was not limiting. Under these conditions, we proposed greater flux through the regulatory steps of the Kreb's cycle would be beneficial. By using partial inhibition of complex I by rotenone, we placed greater demand on complex II to provide for OXPHOS which in turn would make increased IDH and PDH activity more desirable. We postulated this would elicit effects on *omi* gene expression, given that OMI could degrade IDH3A and PDHB. Our results showed a transient silencing of *omi* expression, which was coincident with increased expression of the novel OMI substrates. This suggested conditions where *omi* expression (and potentially OMI activity) was not beneficial, just as the early stage of 6OHDA neurotoxicity suggested a time point when it was.

Further experiments to discern the regulatory players in this altered *omi* expression are required. Interestingly studies investigating the cleavage of CIAP (a member of the IAP family) identified OMI as the serine protease involved, and found expression of *omi* was increased upon activation of the transcription factor p53 [39]. This established *omi* as a downstream target gene of p53 and the presence of a p53 dependent apoptotic pathway in mammals. While this pathway operates in the cytosol and directly impacts cytosolic OMI, it is possible that p53 plays a role in our toxicity studies where the effects are initiated in the mitochondrion and lead to increased *omi* gene expression. However, the transcriptional mechanism of *omi* down-regulation, in our sub-lethal studies, also mediated by the mitochondrial demands, requires greater investigation.

Until this study, the beneficial role of OMI in the mitochondrion has largely been considered as one of maintaining protein quality and turn over. A model recently postulated by Plun-Favreau et al. suggested the mitochondrial kinase PINK1 mediated OMI's activity. In this model PINK1 kinase activity was thought to increase proteolytic capability of OMI, and protect cells from stress-induced mitochondrial dysfunction [40]. The same researchers found PD patients had considerably less OMI in which its key site (OMI S^142^) was phosphorylated by PINK1 [28]. Their model described cells with OMI trimers that contained one or more monomers lacking S^142^ phosphorylation, would be more vulnerable to mitochondrial stress. Additionally they found that idiopathic PD tissue showed greater amounts of phosphorylated OMI compared with normal controls. This implied PINK1 and OMI/HtrA2 operated within a dynamic stress-sensing model, although it could not be determined whether high levels of PINK-activated HtrA2 were a protective response or a cause of nigral cell death. Further to this model is a second protease known as Rhomboid-7 in drosophila and its orthologue PARL in mammals. Whitworth et al discovered Rhomboid-7 was capable of binding both OMI and PINK1, as well as cleaving them in their different intramembrane domains [29]. This provides an additional regulatory means of increasing the mobile pool of both proteins in the intermembrane space. Whether this translates to more or less OMI for cleaving IMS or membrane-associated proteins (respectively), or whether this directly impacts the interaction between OMI and PINK1, remains to be tested. Since PARL is ATP-dependent, where OMI is not, it represents an intriguing aspect of regulation to the PINK1-HtrA2 stress response model.

Our results provide a new piece in this puzzle of describing the role of OMI in the mitochondrion. We believe this protease to be a regulator of IDH3A and PDHB entering the organelle and suggest it is most likely working from a baseline level of activity, so the mitochondrion may be poised to respond according to demand. While OMI is unlikely to be the only regulator, its ability to function independent of ATP should not be overlooked, particularly when key players, such as other mitochondrial proteases, are largely ATP-dependent. Regulation of OMI activity can now be sub-divided between autocatalysis (from inactive to active), PINK1 phosphorylation (from baseline to activated) and Rhomboid-7 modification (from membrane to IMS localization). Our model adds further to the PINK1-HtrA2 stress-sensing model. We suggest, as part of the stress-sensing model, that a pool of deficient or over-active OMI would be deleterious when there is a need to switch between OXPHOS and substrate-level phosphorylation, whether this may be due to a need to avoid excessive ROS production, or a need to generate more ATP. In the case of too much (or aberrantly activated) OMI, the chaperone role of HSPA8 would be destroyed, which could potentially limit PDH and mtIDH activity. This situation may also precipitate the re-location of OMI into the cytosol along with other toxic cell changes, depending upon the type of cell, its metabolic needs, and the duration of the demands placed upon it. In the case where OMI activity was absent or unable to be activated (due to PINK1 deficiencies) during conditions of less demand (but when substrates were plentiful), there would be little or no protease capable of controlling IDH3A and PDHB import. With one less control mechanism, the continued activity and pressure on OXPHOS would generate greater ROS accumulation. Two such paradigms exemplifying the second case may present as #1. fast-firing, dopaminergic neurons (with their need to rapidly restore conditions after repetitive, sequential firing of action potentials) or #2. the developing *mnd*2 mouse-pup. The former situation is further complicated by calcium ion fluctuations to levels capable of activating both of these Kreb's cycle enzymes, which would exacerbate the pressure on OXPHOS. Under these conditions, PINK1 would have a positive role in maintaining OMI activation, and subsequently its substrates greater degradation. The role of Rhomboid-7 requires greater scrutiny in our model. However, it is tempting to suggest that Rhomboid-7 processing would rid the mitochondrial membrane of its tethered OMI pool, which could only be replenished by newly synthesized OMI. As such, over or under-active Rhomboid-7 would impact our model in an ATP-dependent manner.

Several key experiments still need to be addressed, including determining the HSPA8, IDH3A and PDHB levels and expression patterns in the *mnd*2 developing mouse brain. Our current research also focuses on establishing a cellular assay in which the manipulation of OMI activity and its impact on Kreb's cycle function and ROS accumulation can be measured. We include the use of Omi phosphomutants to further establish the effect of PINK1 activation of OMI on our novel substrates. Likewise future studies with OMI mutants unable to be cleaved by Rhomboid-7/PARL could further improve our understanding of the involvement of mitochondrial OMI in the pathogenesis of PD.

## Materials and Methods

### Cell culture, transfection and isolation of mitochondria

Cells (HEK-293 and SH-SY5Y, ATCC) were grown in 10% FBS/DMEM supplemented with 20 U/mL penicillin and 20 ug/mL streptomycin at 37°C in 5% CO_2_/95% air. Small wells were transfected using lipofectamine following the manufacturer's protocol. Cells on large plates (150 cm) were transfected using the Calcium Phosphate method [41]. Detachment of cells was achieved with 10 mM EDTA treatment, followed by centrifugation and resuspension of the pellet in mitochondrial isolation medium (1 mM EDTA, 10 mM HEPES, 250 mM sucrose, pH 7.4). Cells were permeabilised using digitonin (400 ng/mL) incubation and then ruptured by homogenisation in a Dounce glass homogenisor. Cell debris was removed by centrifugation at 5000 Xg. The supernatant was centrifuged at 12000 Xg to form a crude mitochondrial pellet. Protein estimations were done using the BCA protein assay kit (Pierce #23225). Isolated mitochondria were divided into two equal parts and incubated in reaction medium (250 mM sucrose, 10 mM MOPS, 10 mM KH_2_PO_4_, 5 mM MgCl_2_ @ pH 7.2) at room temperature with gentle agitation to ensure oxygenation. Each part received either inhibitor (ucf-101) or equal volume DMSO. The inhibitor ucf-101 (EMD Chemicals #496150) was dissolved in DMSO. Commercially available recombinant HtrA2 (#1458-HT) was purchased from R&D Systems and digestion studies were done according to the manufacturer's protocol.

### Protein separation and detection

Mitochondrial proteins were solubilised via freeze-thaw repeats in the presence of 1% Triton X-100. Insoluble matter was removed by centrifugation. IEF was performed on a Protean BioRad according to the manufacturer's instructions. Proteins that required concentration were done so using a PALL 3K mini-column. PAGE was performed after IEF on Criterion pre-cast IPG-gels. Gels were silver stained using SilverSNAP stain for Mass Spectrometry (Pierce #24600) and quantified using ImageJ (included as supplementary information S1). Separation via 1D PAGE was performed on Invitrogen pre-cast gels with Western transfer onto PVDF membrane using either a Criterion or Invitrogen blotter. All immunoblots were blocked in 2% BSA. Antibodies were purchased from Sigma (anti-FLAG #F7425), R&D systems (anti-Omi/HtrA2 #AF1458), Molecular Probes (anti-PDHB #A-21323), Everest (anti-HSPA8 #EB07295) and (Abgent anti-IDH3A #AP1927). Fluorescent secondary antibodies were purchased from Molecular Probes (#A11001, A21428, A21235 or A21432). HRP-conjugated secondary antibodies were purchased from Pierce (#18584) or Amersham (NA934V). FLAG-tagged OMI monomers were immunoprecipitated using EZview Red Anti-FLAG Affinity Gel beads (Sigma #A2220). Endogenous HSPA8 was immunoprecipitated using Sepharose Protein A beads (Sigma #P3391) to which the antigen-anti-HSPA8 antibody complexes were bound, according to the manufacturer's protocol with modified centrifuge speeds which were kept below 4,000 Xg. HA-tagged HSPA8 was immunoprecipitated using EZView Red Anti-HA affinity gel (Sigma#E6779) and eluted using Influenza hemagglutinin (HA) peptide (Sigma#I2149) according to the manufacturer's protocol with the exception of the centrifuge speeds as stated previously. The size (approximately 61 kDa) of the HA.HSPA8 corresponded to isoform 2 with a short additional pre-sequence (from the UTR) and the HA sequence.

### RNA extraction, cDNA synthesis and qPCR

Total RNA was extracted from cells using the TriZOL method (Invitrogen #15596-026). Synthesis of cDNA from 1 ug of RNA_t_ was achieved using the iScript kit (BioRad #170-8891). Analysis of gene expression was performed on an ABI Prism7000 with CYBR Green and gene specific primers. All results were normalized to β-actin, before being expressed as a percentage of the control (untreated) sample. Concentrations of toxins were chosen from our previous studies.

## Supporting Information

Supplementary Information S1 All spots were corrected for background using the integrated density (ImageJ software). In Table S1 and S2 LDH and Spot #4, of the (-) ucf-101 and OMI.S306C/G399S gels respectively, were < background, denoted here as “neg”. Control spots were chosen arbitrarily to compare overall loading densities. In all cases the FLAG.OMI.S306C control spots were less concentrated than those in OMI double mutant samples, implying the intensity of the identified spots was under-estimated by approximate factor of 2 to 2.5X. The (-) ucf-101 control spots were slightly greater or slightly less concentrated than their equivalent in the (+) ucf-101.
(0.02 MB DOC)
