# Supplementary material for: Novel Mitochondrial Substrates of Omi Indicate a New Regulatory Role in Neurodegenerative Disorders
Source: PLoS One. 2009 Sep 18;4(9):e7100. doi: 10.1371/journal.pone.0007100 (PMC2740829; doi:10.1371/journal.pone.0007100)
Supplement: Supplementary Information S1 — All spots were corrected for background using the integrated density (ImageJ software). In Table S1 and S2 LDH and Spot #4, of the (-) ucf-101 and OMI.S306C/G399S gels respectively, were < background, denoted here as “neg”. Control spots were chosen arbitrarily to compare overall loading densities. In all cases the FLAG.OMI.S306C control spots were less concentrated than those in OMI double mutant samples, implying the intensity of the identified spots was under-estimated by approximate factor of 2 to 2.5X. The (-) ucf-101 control spots were slightly greater or slightly less concentrated than their equivalent in the (+) ucf-101. (0.02 MB DOC) [file pone.0007100.s001.doc]

Table S1: Quantification of +/- ucf-101 2D silver-stained gel (figure 1)

|  | **(+) ucf101:(-)ucf101**  **Integrated densities**  **(corrected for background)** |
| --- | --- |
| HSPA8 | 13.8 |
| LDH | neg. |
| IDH3A | 2.4 |
| PDHB | 3.9 |
| **control spots** |  |
| 1 | 1.2 |
| 2 | 1.4 |
| 3 | 0.7 |

Table S2: Quantification of entrapment 2D gel (figure 2)

|  | **OMI.S306C:OMI.S306C/G399S**  **Integrated densities**  **(corrected for background)** |
| --- | --- |
| HSPA8 | 1.7 |
| F10 | 3.8 |
| F11 | 58.0 |
| Erp57 | neg. |
| **control spots** |  |
| 1 | 0.6 |
| 2 | 0.5 |
| 3 | 0.4 |
